# Effects of plastic film mulching on soil microbial carbon metabolic activity and functional diversity at different maize growth stages in cool, semi-arid regions

**DOI:** 10.3389/fmicb.2024.1492149

**Published:** 2024-10-28

**Authors:** Meng Kong, Ming-Jing Huang, Zhi-Xian Zhang, Jiang Long, Kadambot H. M. Siddique, Dong-Mei Zhang

**Affiliations:** ^1^Shanxi Province Key Laboratory of Sustainable Dryland Agriculture, Shanxi Institute of Sustainable Dryland Agriculture, Shanxi Agricultural University, Taiyuan, China; ^2^Key Laboratory of Sustainable Dryland Agriculture (Co-construction by Ministry of Agriculture and Rural Affairs and Shanxi Province), Shanxi Agricultural University, Taiyuan, China; ^3^Institute of Agriculture, The University of Western Australia, Perth, WA, Australia

**Keywords:** soil microbial community, soil quality, plant growth, crop yield, soil carbon

## Abstract

**Introduction:**

Plastic film mulching has been widely used to enhance soil hydrothermal conditions and increase crop yields in cool, semi-arid areas. However, its impact on soil microbial carbon metabolic activity and functional diversity during plant growth remains unclear despite their important roles in nutrient cycling and soil quality evaluation.

**Methods:**

This study used the Biolog EcoPlate technique to investigate the dynamics and driving factors of soil microbial carbon metabolic activity and functional diversity at different maize growth stages following plastic film mulching.

**Results and discussion:**

The results revealed that film mulching significantly increased microbial carbon metabolic activities [represented by average well color development (AWCD)] by 300% at the seedling stage and by 26.8% at maturity but decreased it by 47.4% at the flowering stage compared to the control (without mulching). A similar trend was observed for the microbial functional diversity index. Redundancy analysis identified soil moisture (SM), soil temperature (ST), dissolved organic carbon (DOC), microbial biomass carbon (MBC), and bacteria amounts as the primary factors influencing changes in soil microbial carbon source utilization. The mulch treatment significantly increased SM at all growth stages, while its warming effect disappeared at the flowering stage. Soil DOC, MBC, and bacterial populations were notably higher under mulching at the seedling and maturity stages but lower at the flowering stage. Pearson correlation analysis showed that changes in SM, ST, DOC, MBC, and bacterial populations positively correlated with the utilization of all carbon source classes, AWCD, and functional diversity indexes after film mulching. Furthermore, maize grain yield and water use efficiency increased by 142 and 129%, respectively, following film mulching. In conclusion, plastic film mulching enhanced soil microbial carbon metabolic activity and functional diversity at the seedling and maturity stages, improving crop yields in cool, semi-arid areas. Furthermore, the decrease in soil carbon metabolic capacity at flowering stage highlights that supplementing soil carbon sources should be considered after continuous film mulching to sustain or enhance farmland productivity and soil quality.

## Introduction

Low temperatures and drought significantly hinder crop growth and development in cool, semi-arid regions, leading to reduced yields (Wang et al., 2016a; Kong et al., 2020). Plastic film mulching has been widely adopted in these areas to address the growing demand for food and ensure food security (Zhou et al., 2009; Zhang et al., 2018; Li et al., 2020). This technique involves covering the soil with plastic film to intercept rainwater, which then infiltrates through seeding holes. This process reduces water and heat exchange between the soil and air, ultimately reducing soil water evaporation, improving soil hydrothermal conditions and water and thermal use efficiencies, promoting crop growth, and increasing crop yields (Zhou et al., 2009; Liu et al., 2014b; Kong et al., 2020). However, continuous plastic film mulching can lead to excessive nutrient depletion due to increased crop uptake, potentially reducing soil fertility, especially in areas with limited nutrient availability and organic matter (Gan et al., 2013; Wang et al., 2014; Steinmetz et al., 2016; Zhang et al., 2017). Therefore, concerns have arisen regarding the long-term effects of plastic film mulching on soil quality, which directly impacts its viability as a sustainable agricultural practice.

Soil microorganisms play a crucial role in farmland soil ecosystems, including organic matter decomposition, nutrient transformation, soil structure formation, and energy flow. They also mediate plant–soil interactions, key indicators of soil quality (Liu B. et al., 2022). As such, changes in the soil microbial community directly or indirectly influence crop growth and yield. Given the altered soil environment under film mulching, shifts in soil microorganisms can be expected (Li et al., 2022b). For instance, Luo et al. (2019) reported that film mulching decreased total phospholipid fatty acids (PLFA) and the absolute abundance of fungi, bacteria, and actinomycetes. Similarly, Huang et al. (2019) found that film mulching increased bacterial diversity and the relative abundance of *Proteobacteria* but decreased the abundance of *Actinobacteria*. Wan et al. (2023b) observed that film mulching altered fungal community structure, reduced plant pathogens, and did not affect fungal diversity. While previous research has primarily focused on structural diversity—changes in species composition, key taxa, and abundance—the functional diversity of soil microorganisms remains underexplored. Functional diversity reflects the ecological roles of soil microorganisms, such as nutrient cycling and organic matter decomposition, making it a critical indicator for assessing changes in soil quality (Wang et al., 2022). Soil microbial carbon source utilization has been widely regarded as a crucial indicator for evaluating the functional diversity of microorganisms (Gao et al., 2024; Huang Z. et al., 2024; Huang T. et al., 2024). Studying how soil microbial carbon metabolic activity and functional diversity respond to continuous plastic film mulching is essential for evaluating soil quality and predicting soil fertility trends. Soil microbial carbon metabolic activity and functional diversity can be assessed using the Biolog EcoPlate method by comparing the pattern of the carbon source utilization of different microorganisms (Huang Z. et al., 2024). It has been widely used in microbial community studies as a simple and rapid method (Li et al., 2021; Zhang et al., 2019; Zhang et al., 2021; Zhu et al., 2018).

Soil microorganisms are highly sensitive to environmental changes (Wang et al., 2024). The impact of plastic film mulching on the soil environment—including soil moisture, temperature, organic matter, and nutrients—varies across different growth stages. For example, the warming effect of mulching decreases over time due to crop shading (Zhou et al., 2009; Wang et al., 2016b), and mulching may reduce soil moisture and nutrient levels in the middle and later stages due to increased biomass and water consumption (Liu et al., 2014b; Wang et al., 2014). Consequently, the effects of mulching on soil microbial communities will likely vary across growth stages, influencing soil nutrient cycling and crop growth differently at each stage. However, most studies have focused on specific developmental stages (e.g., flowering or maturity), limiting our understanding of plastic film mulching’s overall impact on soil microorganisms and crop growth.

To address this gap, we relied on a long-term positioning experiment (2013–2018) to investigate how soil microbial functional diversity responds to plastic film mulching across different developmental stages using the Biolog EcoPlate technique. We hypothesized that plastic film mulching increases soil microbial metabolic activity and functional diversity but that this effect would diminish or disappear later in the growth period due to decreased soil moisture, temperature, and nutrient availability. The findings of this study will provide a theoretical foundation for improving farmland productivity and soil quality in cool, semi-arid regions.

## Materials and methods

### Experimental site description

The experiment was started in 2013 at the Dryland Agro-Ecosystem Research Station of Lanzhou University, Zhonglianchuancun village, Yuzhong country, Gansu Province, northwestern China (36°02′ N, 104°25′ E, altitude 2,400 m). The region experiences a cool, temperate, semi-arid climate, with a mean annual air temperature of 6.5°C and annual precipitation of 320 mm. Groundwater is not a viable irrigation source due to its depth, making rainfall the sole water source for crop growth. The soil is classified as Heima soil (Calcic Kastanozem) with a bulk density of 1.28 g cm^−3^, organic carbon of 10.11 g kg^−1^, total nitrogen of 0.71 g kg^−1^, and total phosphorus of 0.77 g kg^−1^.

### Experimental design and field management

The field experiment comprised two treatments: plastic film mulching (mulch) and no mulching (control). A randomized block design with three blocks was used, with each plot covering 40 m^2^ (8 m × 5 m). In the mulch treatment, transparent polyethylene film (0.008 mm thick, 120 cm wide) was applied after removing the previous year’s film and before plowing. All plots were fertilized with 150 kg ha^−1^ nitrogen and 25 kg ha^−1^ phosphorus annually. Every year, maize (cv. Kenyu 10) was sown at 6 cm depth in late April, with a plant spacing of 40 cm and row spacing of 60 cm, using a mechanical dibbler (Yongfeng Agricultural and Forestry Tools Co., Ltd., China). Two weeks after sowing, the holes in the plastic film were enlarged to allow seedlings to emerge and then covered with soil. Rainwater was directed into the soil through these seeding holes. Maize was harvested in early October each year and the straw was removed from the field to feed livestock. Grain yield (dry weight) was determined by harvesting two central rows from each plot. In the current study, we collected the soil and plant samples during the growing season of 2018 (about 6 years after mulching) for the analysis of soil microbial carbon metabolic activity and functional diversity.

### Sampling and measurements

#### Shoot nitrogen (N) and phosphorus (P) uptake

The shoot for determined shoot N and P uptake was sampled after maize harvesting on 8 October 2018. Three representative maize shoots per plot were oven-dried at 75°C, ground, and digested with H_2_O_2_-H_2_SO_4_ to determine shoot N concentration using the Kjeldahl method and shoot P concentration using molybdenum-antimony anti-spectrophotometry (Lu, 2000). Shoot nutrient uptake was calculated as grain yield multiplied by nutrient concentration.

#### Water use efficiency (WUE) and precipitation use efficiency (PUE)

Soil moisture content (SM) was measured at sowing and harvest in each plot in 2018. Soil samples were collected at 20 cm intervals to 200 cm depth using an 8 cm diameter soil auger, then oven-dried at 105°C to constant weight. Soil water storage (SWS) (Equation 1) and evapotranspiration (ET) (Equation 2) were calculated as follows:


(1) SWS=SM×SBD×SH×0.1

(2) $$ET=SWS_{s}-SWS_{h}+P$$

where SM is soil moisture content (%), SBD is bulk density (g cm^−3^), SH is layer thickness (cm), $SWS_{s}$ and $SWS_{h}$ are soil water storage at the sowing and harvest, respectively (mm), and P is precipitation (mm) during the growing season.

Water use efficiency (WUE) (Equation 3) and precipitation use efficiency (PUE) (Equation 4) were calculated as follows:


(3) WUE=Y/ET

(4) PUE=Y/P

where Y is grain yield (kg ha^−1^), ET is soil water consumption (mm), and P is precipitation during the growing season (mm).

#### Plant biomass and soil properties

Plant and soil samples were collected at the seeding, flowering, and maturity stages during 2018. Three plants were randomly uprooted (60 length, 40 cm width, and 60 cm deep) using a hand-held shovel. Shoots and roots were separated and oven-dried at 105°C for 30 min, then at 75°C to constant weight. Topsoil (0–20 cm) samples were collected from six points in each plot using an 8 cm diameter soil auger and mixed into a composite sample. Soil samples were immediately placed in iceboxes and transported to the laboratory. After passing through a 2 mm soil sieve, samples were divided into three parts: one part was used immediately for determining soil dissolved organic carbon (DOC), inorganic nitrogen (IN), microbial biomass carbon (MBC), N (MBN), and P (MBP); one part was dried naturally for measuring soil organic carbon (SOC), total nitrogen (STN), total P (STP), and available P (AP); and one part was stored at 4°C for assessing the soil microbial metabolic functional diversity and the cultural populations of bacteria and fungi within 3 days.

Soil temperature (ST) was measured using a temperature logger (179-UT, Apresys Inc., United States) at a depth of 10 cm. Soil moisture content (SM) was determined by oven-drying at 105°C. SOC, STN, STP, and AP were determined using the Walkley and Black dichromate oxidation method (Lu, 2000), Kjeldahl analysis (Lu, 2000), molybdenum-antimony colorimetric method (Lu, 2000), and Olsen-P method (Olsen et al., 1954), respectively. Soil IN was determined by auto-flow injection analyzer (Skalar, Breda, Netherlands) after extraction with 2 mol L^−1^ KCl. Soil MBC, MBN, and MBP were measured using the chloroform fumigation–extraction method with efficiency constants of 0.45, 0.54, and 0.40 (Jenkinson, 2004), respectively. Soil DOC was determined using a total organic carbon analyzer (Multi N/C 3100, Analytik Jena, Germany) after extraction with 0.5 mol L^−1^ K_2_SO_4_. The amounts of bacteria and fungi were quantified using the soil dilution plating method.

#### Soil microbial carbon metabolic activity and functional diversity

The carbon metabolic activity and functional diversity of the soil microbial community were assessed using the Biolog EcoPlate method (Biolog, Hayward, United States). Each plate contains 31 carbon sources and one blank well with water, categorized into eight classes: amines, amino acids, polymers, phosphorylated chemicals, carbohydrates, carboxylic acids, phenolic compounds, and esters (Bastida et al., 2014). This method is widely used in carbon source metabolic activity and functional diversity studies on soil microbial communities based on the fingerprint of the carbon source utilization of different microorganisms (Li et al., 2021; Zhang et al., 2021; Huang Z. et al., 2024). Briefly, approximately 5 g fresh soil was suspended in 90 mL sterile saline solution (0.85% NaCl) in a 250 mL sterile triangular flask, shaken for 20 min at 200 rpm min^−1^ at 25°C, then allowed to stand at 4°C for 10 min. The supernatant was diluted to a 10^−3^ concentration using a sterile saline solution. The EcoPlates were preheated to 25°C, and 150 μL of the diluted soil suspension was added to each well and incubated at 25°C for 168 h. Absorbance values in each well were quantified at 590 nm using a Multi-Mode Microplate Reader (Varioskan Flash, Thermo Scientific, United States) every 24 h.

The microbial metabolic activities were reflected by average well color development (AWCD). The AWCD represents the overall carbon substrate utilization potential of microbial communities, calculated by Equation 5 (Zhang W. et al., 2022):


(5) $$\mathrm{AWCD}=\frac{\sum \left(A_{i}-C\right)}{31}$$

where *A*_i_ is the absorbance value of each well, and *C* is the absorbance value of the blank well.

Since AWCD increased at a decreasing rate and stabilized after 120 h, the absorbance values at 120 h were analyzed to characterize the metabolic diversity of soil microbial community carbon sources using the McIntosh index (U) and Shannon index (H′), calculated by Equations 6, 7 (Zhang et al., 2021):


(6) $$U=\sqrt{\sum n_{i}^{2}}$$

(7) $$H'=-\sum P_{i}\times \ln P_{i}$$

where $n_{i}$ is the relative absorbance value of well i ( $n_{i}=A_{i}-C$ ) and $P_{i}$ is the ratio of the relative absorbance of well i ( $n_{i}$ ) to the sum of absorbance values of all wells.

### Statistical analysis

One-way analysis of variance was used to evaluate the effects of film mulching on grain yield, WUE, PUE, shoot nutrient uptake, AWCD, McIntosh index, Shannon index, substrate utilization, and soil properties at each growth stage using GenStat v.18.1. Correlations between changes in eight carbon source classes and soil properties were analyzed using Pearson’s correlation coefficient. Redundancy analysis of soil microbial carbon sources utilization and soil properties was performed using Canoco 5.0. All graphs were plotted using Origin Pro 2021.

## Results

### Grain yield, water use efficiency, shoot nutrient uptake, and biomass accumulation

**Figure 1:**
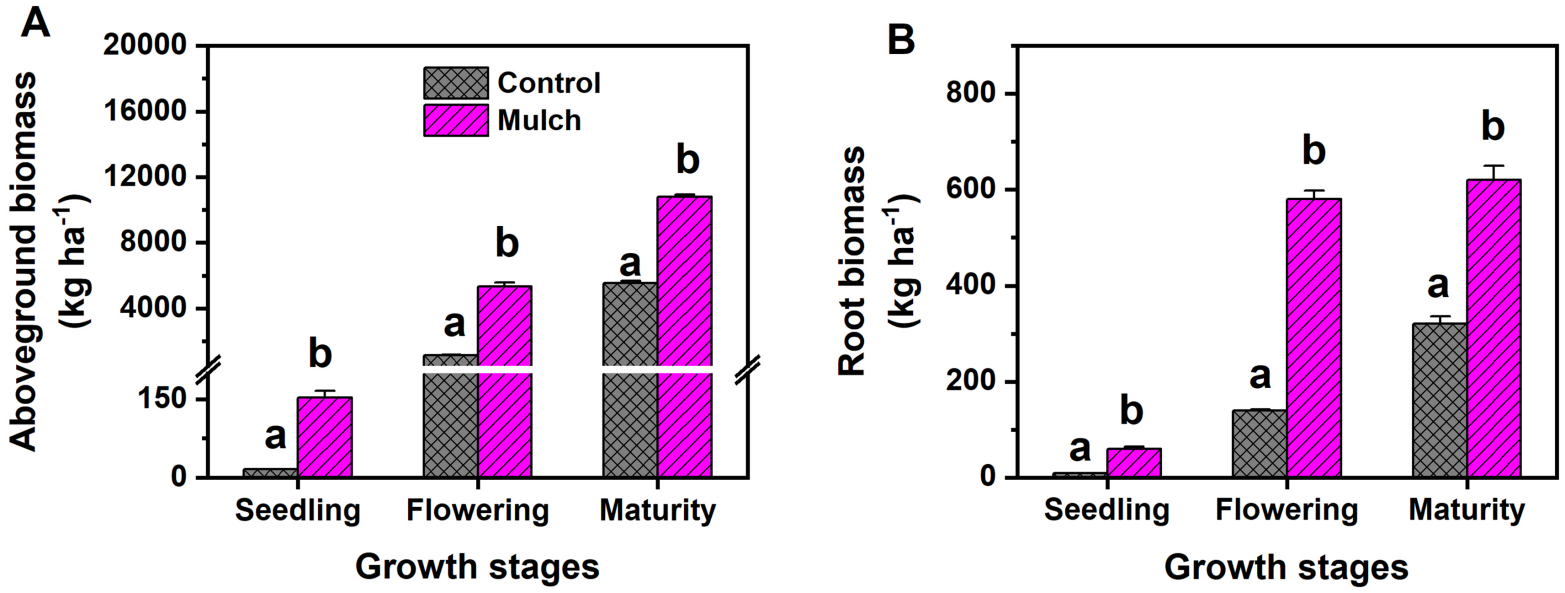
Effects of film mulch on maize (A) aboveground biomass and (B) root biomass at different maize growth stages. Different lowercase letters for the same growth stage indicate significant differences among treatments at *p* < 0.05. The experiment treatments: Mulch, plastic film mulching; Control, no mulching.

Compared to the control, the mulch treatment significantly increased maize grain yield, WUE, and PUE by 142, 129, and 142%, respectively (Table 1). The mulch treatment also had significantly higher shoot N and P uptake than the control (Table 1). Plastic film mulching significantly increased maize biomass, with aboveground and root biomass increasing by 884 and 555% at the seeding stage, 370 and 316% at the flowering stage, 109 and 93% at the maturity stage, respectively (Figure 1).

**Table 1:** Effects of film mulch on maize grain yield, water use efficiency (WUE), precipitation use efficiency (PUE), and shoot N and P uptake.

Treatment	Grain yield (kg ha^−1^)	WUE (kg ha^−1^ mm^−1^)	PUE (kg ha^−1^ mm^−1^)	Shoot N uptake (kg ha^−1^)	Shoot P uptake (kg ha^−1^)
Control	2,285 ± 130 a	6.29 ± 0.30 a	5.96 ± 0.34 a	58.8 ± 1.55 a	11.4 ± 0.41 a
Mulch	5,523 ± 125 b	14.39 ± 0.28 b	14.42 ± 0.33 b	104 ± 3.72 b	20.5 ± 0.70 b

Different lowercase letters in the same years within a column refer to significant differences at *p* < 0.05. The experiment treatments: Mulch, plastic film mulching; Control, no mulching.

### Soil hydrothermal conditions and nutrients

**Figure 2:**
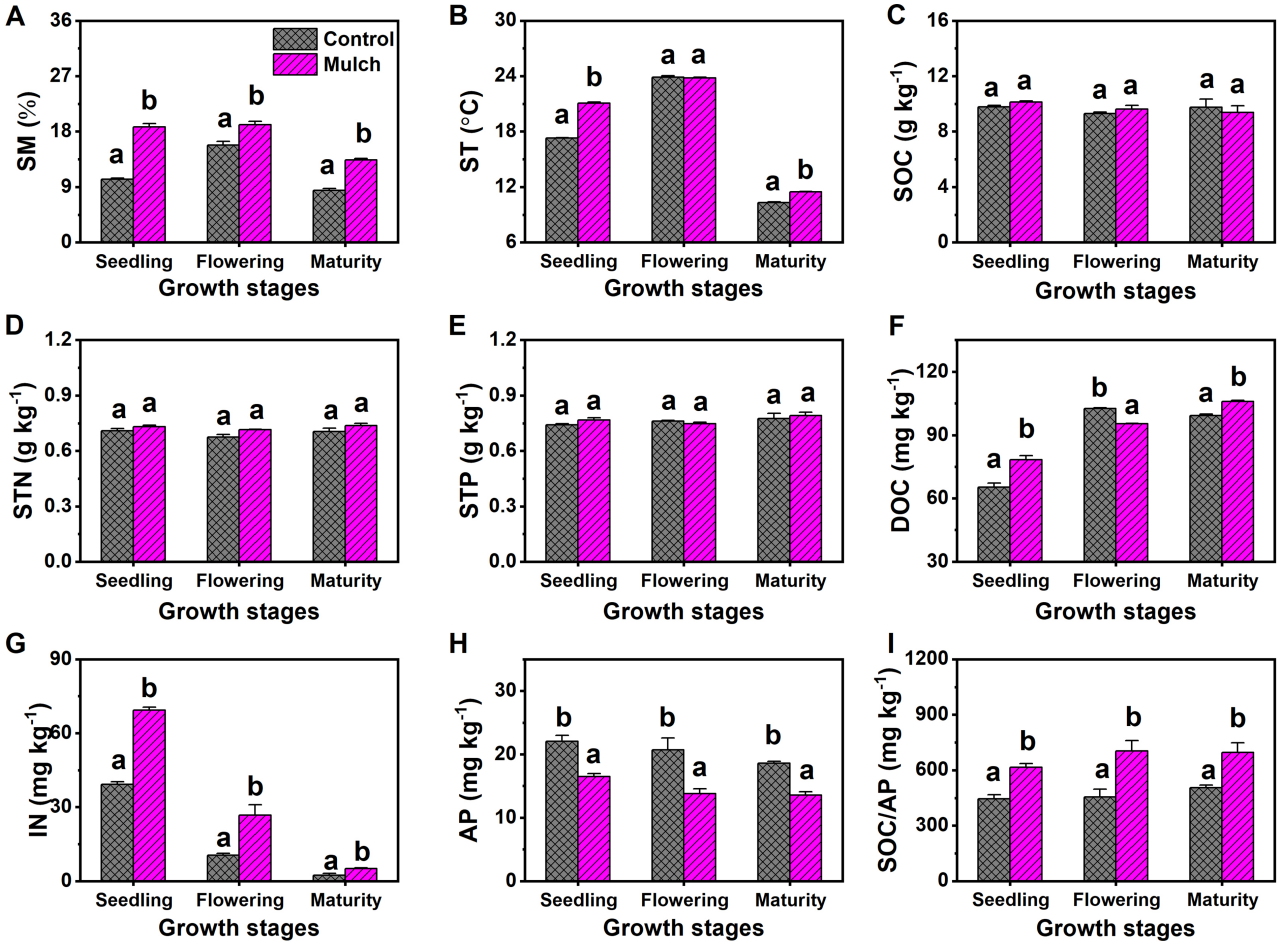
Effects of film mulch on (A) soil moisture (SM), (B) temperature (ST), and the content of (C) organic carbon (SOC), (D) total nitrogen (STN), (E) total phosphorus (STP), (F) dissolved organic carbon (DOC), (G) inorganic nitrogen (IN), (H) available phosphorus (AP), and (I) C:P ratio (SOC/AP) at different maize growth stages. Different lowercase letters for the same growth stage indicate significant differences among treatments at *p* < 0.05. The experiment treatments: Mulch, plastic film mulching; Control, no mulching.

The mulch treatment consistently had higher SM than the control at all growth stages (Figure 2A). Film mulching significantly increased ST by 3.8°C at the seedling stage and 3.4°C at the maturity stage, with no effect observed at the flowering stage (Figure 2B). Film mulching did not significantly affect SOC, STN, or STP, nor did the maize growth stage (Figures 2C–E). Soil DOC increased with plant growth, whereas IN and AP decreased. The mulch treatment had significantly higher soil DOC than the control at the seedling and maturity stages, but significantly lower soil DOC at the flowering stage (Figure 2F). The mulch treatment also had significantly higher soil IN and C:P ratio and lower AP than the control at all stages (Figures 2G–I).

### Soil microbial biomass and cultural microbial amounts

**Figure 3:**
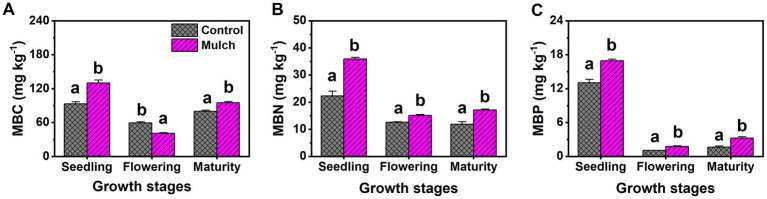
Effects of film mulch on the content of soil (A) microbial biomass carbon (MBC), (B) microbial biomass nitrogen (MBN), and (C) microbial biomass phosphorus (MBP) at different maize growth stages. Different lowercase letters for the same growth stage indicate significant differences among treatments at *p* < 0.05. The experiment treatments: Mulch, plastic film mulching; Control, no mulching.

**Figure 4:**
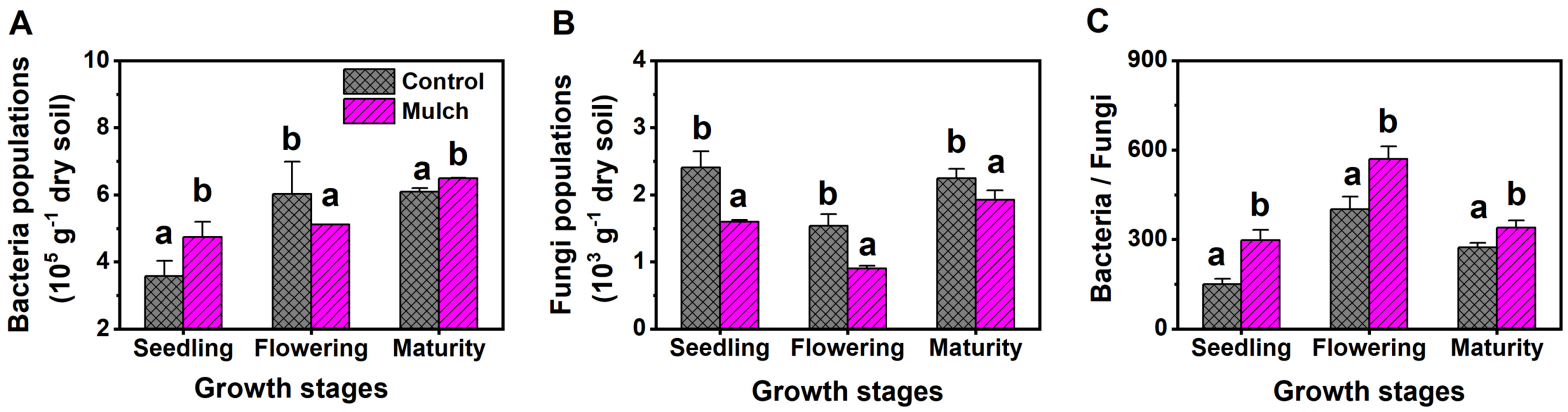
Effects of film mulch on the amounts of (A) bacteria and (B) fungi, and (C) bacteria: fungi ratio at different maize growth stages. Different lowercase letters for the same growth stage indicate significant differences among treatments at *p* < 0.05. The experiment treatments: Mulch, plastic film mulching; Control, no mulching.

Film mulch significantly increased soil MBC and bacterial populations at the seedling and maturity stages but decreased them at the flowering stage (Figures 3A, 4A). The mulch treatment had significantly higher soil MBN, MBP, and bacteria: fungi ratio and lower soil fungal populations than the control at all stages (Figures 3, 4).

### Soil microbial carbon metabolic characteristics

Film mulch significantly increased soil microbial carbon metabolic activities (represented by AWCD) by 300% at the seedling stage and 26.8% at the maturity stage but decreased it by 47.4% at the flowering stage (Table 2). The effects of film mulching on microbial metabolic diversity indices [McIntosh index (U) and Shannon index (H′)] mirrored those observed for AWCD (Table 2).

**Table 2:** Effects of film mulch on average well color development (AWCD) and diversity indices (McIntosh index and Shannon index) of soil microbial communities at different maize growth stages.

Growth stage	Treatment	AWCD	McIntosh index	Shannon index
Seeding stage	Control	0.152 ± 0.002 a	1.563 ± 0.129 a	2.489 ± 0.002 a
	Mulch	0.608 ± 0.005 b	4.045 ± 0.057 b	3.198 ± 0.007 b
Flowering stage	Control	0.517 ± 0.008 b	3.571 ± 0.109 b	3.124 ± 0.019 b
	Mulch	0.272 ± 0.008 a	1.991 ± 0.070 a	3.022 ± 0.016 a
Maturity stage	Control	0.299 ± 0.001 a	2.408 ± 0.014 a	2.861 ± 0.002 a
	Mulch	0.376 ± 0.004 b	2.623 ± 0.028 b	3.096 ± 0.002 b

Different lowercase letters for the same growth stage within a column refer to significant differences at *p* < 0.05. The experiment treatments: Mulch, plastic film mulching; Control, no mulching.

**Figure 5:**
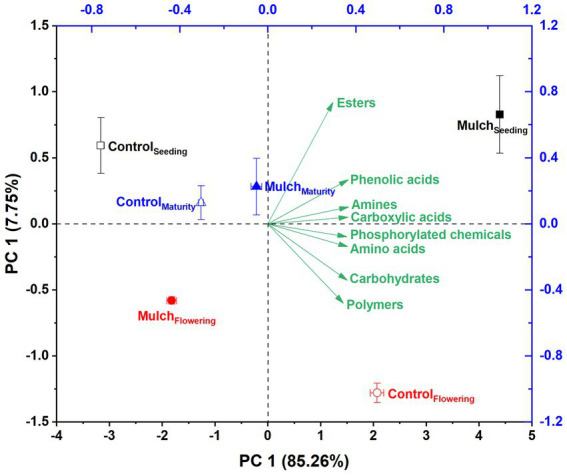
Principal components analysis based on soil microbial carbon source utilization under different mulching treatments at different maize growth stages. The experiment treatments: Mulch, plastic film mulching; Control, no mulching.

**Figure 6:**
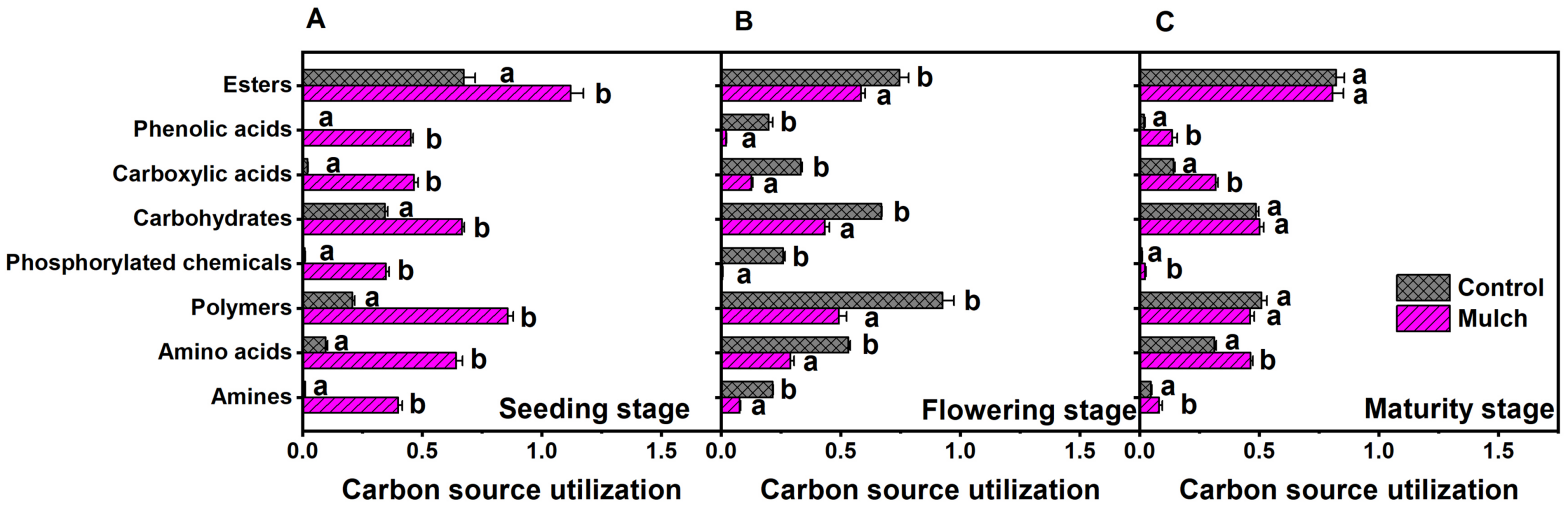
Effects of film mulch on the utilization of eight carbon source classes by soil microbial communities at **(A)** seeding, **(B)** flowering, and **(C)** maturity stages. Different lowercase letters for the same growth stage indicate significant differences among treatments at *p* < 0.05. The experiment treatments: Mulch, plastic film mulching; Control, no mulching.

Principal component analysis revealed distinct differences between mulching treatments and growth stages, with film mulch significantly altering microbial metabolic activities across all stages (Figure 5). PCA explained 93.01% of the variation, with PC1 explaining 85.26% and PC2 explaining 7.75%. The separation between mulching treatments and the control was prominent along the PC1 axis, while the growth stages were distinguished along the PC2 axis (Figure 5). The distance between the mulch and control treatments gradually decreased with plant growth, suggesting a diminishing effect of mulching on microbial metabolic activity over time (Figure 5). Film mulch significantly enhanced the utilization of all carbon sources at the seedling stage but reduced it at the flowering stage (Figure 6). At maturity, film mulch did not affect the utilization of polymers, carbohydrates, or esters but significantly increased the utilization of amines, amino acids, phosphorylated chemicals, carboxylic acids, and phenolic compounds (Figure 6).

### Correlations between soil properties and microbial carbon sources utilization

**Figure 7:**
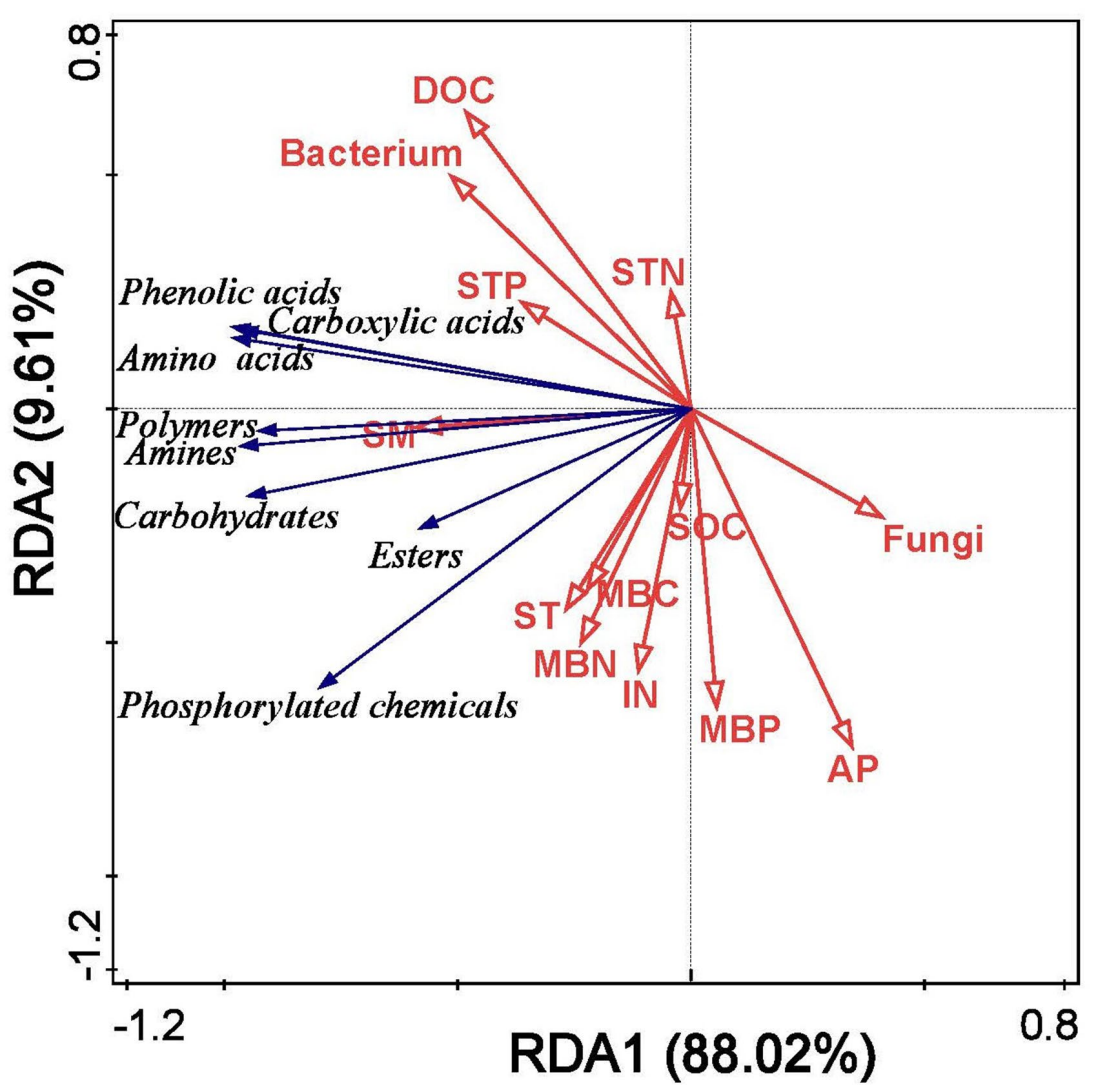
Redundancy analysis of soil microbial carbon source utilization and soil properties under different mulching treatments. SM, soil moisture; ST, soil temperature; DOC, dissolved organic carbon; IN, inorganic nitrogen; AP, available phosphorus; SOC, soil organic carbon; STN, soil total nitrogen; STP, soil total phosphorus; MBC, microbial biomass carbon; MBN, microbial biomass nitrogen; MBP, microbial biomass phosphorus.

Redundancy analysis demonstrated that soil properties effectively explained changes in microbial carbon source utilization under different film mulching treatments, with the first two axes accounting for 97.63% of the total variation (Figure 7). The main factors affecting soil microbial carbon source utilization were SM, ST, DOC, MBC, and bacterial counts (Figure 7).

**Figure 8:**
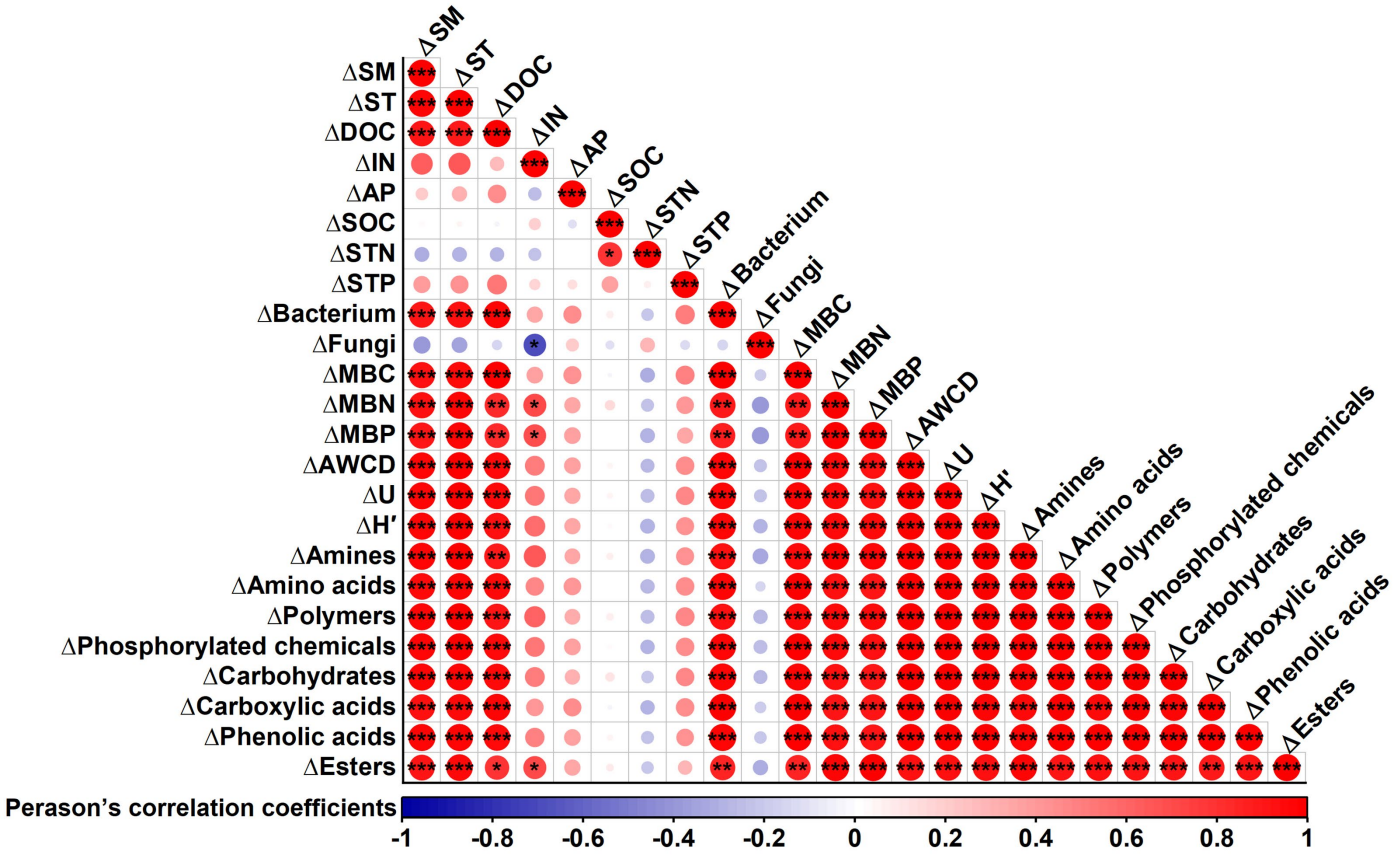
Correlation analysis between the changes in soil microbial carbon metabolic characteristics (carbon source utilization and diversity index) and changes in soil properties after film mulching. ∆ represents changes in soil microbial metabolic characteristics and soil properties after film mulching. Soil properties: SM, soil moisture; ST, soil temperature; DOC, dissolved organic carbon; IN, inorganic nitrogen; AP, available phosphorus; SOC, soil organic carbon; STN, soil total nitrogen; STP, soil total phosphorus; MBC, microbial biomass carbon; MBN, microbial biomass nitrogen; MBP, microbial biomass phosphorus. Soil microbial carbon metabolic characteristics: AWCD, average well color development; U, McIntosh index; H′, Shannon index. ***p ≤ 0.001, **p ≤ 0.01, *p ≤ 0.05.

Correlation analysis revealed significant positive correlations between changes in SM, ST, DOC, bacterial counts, MBC, MBN, and MBP with changes in AWCD, McIntosh index, Shannon index, and the utilization of all carbon source classes under film mulching (Figure 8). However, changes in soil IN, AP, and fungal counts showed no significant correlations with these parameters (Figure 8).

## Discussion

Plastic film mulching is widely used to increase soil temperature and moisture, reduce soil water loss through evaporation, and suppress weeds and pathogens, ultimately enhancing crop yields and economic benefits (Liu et al., 2014b; Wan et al., 2023b; Gao et al., 2024; Huang T. et al., 2024). A global meta-analysis revealed that plastic film mulching increases maize yield by an average of 36% compared to non-mulched controls (Zhang K. et al., 2022). Our study found that the mulch treatment increased maize yield by 142%, exceeding the global average and suggesting that film mulching has a more pronounced impact in hydrothermally limited areas. This effect is attributed to the typically low soil temperatures and moisture in such areas, which hinder maize growth and yield. Film mulching significantly improves these conditions, enhancing maize yield. The yield response to mulching diminishes with increased precipitation and temperature (Zhang et al., 2018; Zhang K. et al., 2022). Apart from hydrothermal effects, increased soil nutrient availability contributes to the yield benefits of mulching. Mulching affects soil nitrogen cycling, enhancing microbial abundance, urease, and protease activities, and nitrogen mineralization and inhibits ammonia volatilization, leading to increased soil IN content (Li et al., 2004; Li et al., 2022a; Dou et al., 2023). As maize is a nitrogen-intensive crop, the increased nitrogen availability under mulching supports its growth and biomass accumulation (Wang et al., 2014). However, the mulch treatment had significantly lower soil AP than the control—as reported elsewhere (Ma et al., 2018; Zhu et al., 2018; Cheng et al., 2020; Kong et al., 2023; Zhang N. N. et al., 2023)—due to maize absorption and competition with soil microbes, as evidenced by the increased shoot P uptake, soil C:P ratio, and MBP content (Gu et al., 2018).

Recently, concerns have been raised about the drawbacks of plastic film mulching (Steinmetz et al., 2016), such as environmental pollution from residual film and the long-term effects on soil quality and fertility (Ma et al., 2018). Soil microorganisms, crucial for biogeochemical cycling, are sensitive indicators of soil health and nutrient cycling (Wang et al., 2022; Wang et al., 2024). The impact of plastic film mulching on soil microbial carbon metabolic activity and functional diversity is poorly understood. Our study revealed that the effects of mulching on microbial carbon metabolic activity and functional diversity varied with maize growth stages and were influenced by SM, ST, DOC, MBC, and the bacterial population.

Soil moisture and temperature significantly impact microbial activity (Cui et al., 2020). Increasing soil moisture and temperature within certain ranges can enhance soil microbial metabolic processes by improving substrate and nutrient availability and promoting microbial growth and reproduction (Zhang et al., 2019; Cui et al., 2020; Wang et al., 2024). Our results showed that increased SM and ST under mulching positively correlated with microbial carbon source utilization and functional diversity indexes at the seedling and maturity stages. However, at the flowering stage, the warming effect of mulching diminished due to crop (Zhou et al., 2009), and the water conservation effect decreased with increased biomass and water consumption (Liu et al., 2014b). When warming was less than 1.1°C and water conservation was less than 4.8%, film mulching did not enhance microbial activity (Supplementary Figure S1). Thus, mulching did not increase AWCD, McIntosh, and Shannon indices at flowering stage. The results indicate that the efficacy of film mulching on microbial vitality and diversity largely depends on its warming and water conservation effects.

Soil organic carbon is a key substrate for microbial growth and directly affects microbial activity and diversity (Huang et al., 2008; Huang Z. et al., 2024). In our study, mulching did not significantly affect SOC during the growing period, consistent with some previous research (Liu et al., 2014a; Wang et al., 2016a; Liu et al., 2019). However, other studies report increases (Mo et al., 2020; Liu et al., 2023; Yang et al., 2024) and decreases (Ma et al., 2018; Zhang K. et al., 2023) in SOC due to mulching. Factors like fertilization practices, straw management, soil fertility, treatment duration, and environmental conditions likely influence these conflicting results. Indeed, SOC changes have been linked to the balance between soil organic matter input and mineralization (Wang et al., 2016a). Mulching can promote SOC mineralization through increased microbial activity and enzyme production (Wang et al., 2014; Zhang F. et al., 2022) while enhancing SOC inputs via increased aboveground and root biomass production (Mo et al., 2020). Unlike SOC, film mulching significantly affected soil DOC and MBC. These fractions are active, sensitive indicators of soil organic matter and microbial utilization (Tian et al., 2015; Liu et al., 2019; Gao et al., 2024). The higher the soil DOC and MBC content, the higher the AWCD and functional diversity indexes. At the seedling stage, improved soil hydrothermal conditions under mulching enhance microbial growth and reproduction and SOC mineralization, leading to higher DOC and MBC (Wang et al., 2014) and subsequently increased microbial utilization of carbon sources and diversity indexes (AWCD, H′, and D). As plant growth progresses, labile substrates are depleted (Zhang F. et al., 2022), reducing DOC and MBC (Wang et al., 2014; Wan et al., 2023a) and lowering microbial activity and diversity at flowering. During later growth, mulching increased organic carbon and nutrient inputs from plant residues and rhizodeposits, leading to higher DOC and MBC and enhanced microbial activity.

Soil microorganisms are integral to nutrient cycling, directly influencing nutrient activation, transformation, and transportation. An increase in microbial populations can significantly enhance carbon metabolic activity in the soil (Liu S. et al., 2022). This study found that film mulching significantly impacted microbial populations, driven largely by the quantity and quality of organic matter and soil hydrothermal conditions. Bacteria, which typically use easily decomposed organic matter, benefit from improved hydrothermal conditions (Cao et al., 2011). Thus, the mulch treatment had significantly higher bacterial populations than the control at the seedling and maturity stages—but lower at the flowering stage—associated with changes in ST, SM, DOC, and MBC, ultimately leading to changes in microbial metabolic characteristics. Accordingly, changes in AWCD and functional diversity indexes positively correlated with changes in bacterial populations following mulching. With the growth of plants and environmental changes, soil microorganisms will gradually adapt to the environment, resulting in a narrowing gap in bacterial populations between mulch and control treatments with maize growth stages. This will also affect the microbial community structure, as mulching only affects the utilization of five carbon source classes at the maturity stage, unlike at the seedling and flowering stages. Therefore, the impact of mulching on microbial metabolic activity gradually weakens over time. Additionally, mulching significantly decreased fungal populations and the bacteria: fungi ratio at all growth stages, indicating a shift in the soil microbial community toward bacterial dominance, as reported elsewhere (Zhu et al., 2018; Luo et al., 2019). This shift is crucial because soil dominated by bacteria, with faster organic matter decomposition and higher nitrogen mineralization rates, better supports the rapid nutrient demands of growing crops (Cao et al., 2011).

Higher soil microbial carbon metabolic activity and diversity correlate with increased nutrient supply capacity and crop yields (Liu S. et al., 2022; Zhang W. et al., 2022). However, it is notable that plastic film mulching significantly reduced the utilization of all carbon sources by soil microorganisms at the flowering stage, which implies a decrease in soil carbon metabolism and nutrient supply. Since the flowering period is critical for nutrient demand in maize, this decline could potentially diminish the yield-increasing effects of film mulching. The key reason behind this reduction was decreased available active carbon sources. Therefore, enhancing microbial carbon metabolic activity during the flowering period by supplementing carbon sources is crucial to meet the nutrient needs of maize. Studies have shown that organic fertilizer application and straw returning can rapidly supply easily decomposable organic carbon, fueling the rapid growth and reproduction of microorganisms, thereby enhancing microbial carbon metabolic activities (Yuan et al., 2019). Moreover, these practices can offset the reduction in soil DOC or SOC caused by continuous film mulching, alleviating concerns about the sustainability of farmland productivity (Li et al., 2019; Wu et al., 2024). Therefore, integrating organic fertilizer application and/or straw returning into continuous film mulching systems should enhance microbial activity and further extend the yield benefits of film mulching.

This study used the Biolog method to elucidate the dynamics of soil microbial carbon metabolic activity, functional diversity, and the driving factors influenced by film mulching. The findings provide a scientific basis for the sustainable development of film-mulched farmland. However, some limitations were noted. The Biolog method does not provide detailed information on the composition and structure of functional soil microorganisms (Li et al., 2021; Huang Z. et al., 2024). Therefore, future studies should incorporate metagenomic sequencing, mass spectrum, and nuclear magnetic resonance to explore the effects of mulching on soil microbial functional diversity, specifically focusing on soil microbial community structure, nutrient cycling genes, metabolic pathways, and the molecular structure of soil organic carbon and microbial metabolites. Moreover, further research is needed to investigate the effects of different mulching durations (such as 0, 3, 5, 10, 15, 20, and 30 years) on soil microbial functional diversity and its relationship with soil quality over time.

## Conclusion

Plastic film mulching significantly influenced soil microbial carbon metabolic activity and functional diversity across different growth stages by altering the soil environment. At the seedling and maturity stages, film mulching significantly improved hydrothermal conditions, increased DOC and MBC, enhanced bacterial populations, soil microbial metabolic activity, and carbon metabolic functional diversity, increasing maize growth, nutrient absorption, WUE, PUE, and grain yield. However, at the flowering stage, the effects of mulching diminished as warming and water conservation weakened, leading to a reduction in DOC, MBC, and bacterial populations, resulting in significantly lower soil microbial carbon metabolic activity and functional diversity, suggesting that continuous film mulching may adversely affect soil quality. Therefore, we recommend supplementing soil carbon sources, such as organic fertilizer application and/or straw returning, following continuous mulching. This approach can help maximize the benefits of mulching and promote sustainable agricultural development in cool, semi-arid regions.

## Data Availability

The original contributions presented in the study are included in the article/Supplementary material, further inquiries can be directed to the corresponding authors.
