# Supplementary material for: Effects of plastic film mulching on soil microbial carbon metabolic activity and functional diversity at different maize growth stages in cool, semi-arid regions
Source: Front Microbiol. 2024 Oct 28;15:1492149. doi: 10.3389/fmicb.2024.1492149 (PMC11550987; doi:10.3389/fmicb.2024.1492149)
Supplement: Supplementary file 1 [file Presentation_1.pdf]

Supplementary Material

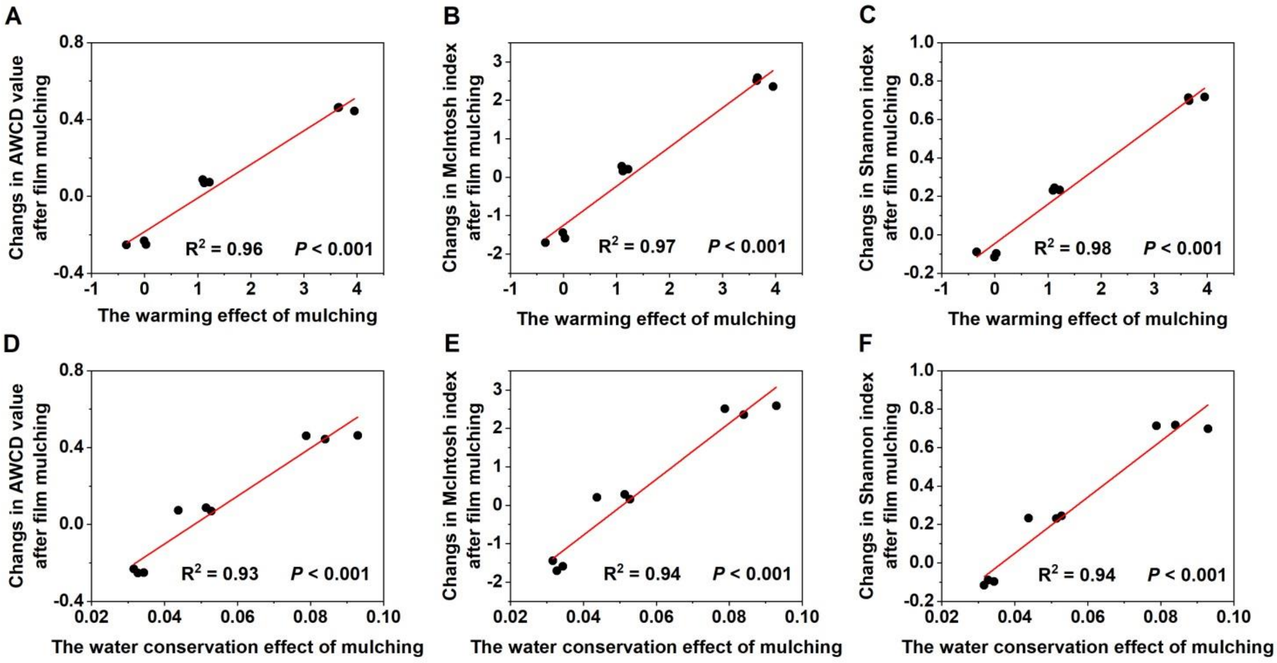

Fig. S1 Relationship between increases in soil temperature and water content and changes in AWCD and soil microbial diversity indexes (McIntosh and Shannon indexes) after mulching.
